# Functional Axis of PDE5/cGMP Mediates Timosaponin-AIII-Elicited Growth Suppression of Glioblastoma U87MG Cells

**DOI:** 10.3390/molecules28093795

**Published:** 2023-04-28

**Authors:** Ya-Fang Liao, Hui-Jun Pan, Nuerziba Abudurezeke, Chun-Lu Yuan, Yan-Li Yuan, Shu-Da Zhao, Dan-Dan Zhang, Shuang Huang

**Affiliations:** 1 Institute of Interdisciplinary Integrative Medicine Research, Shanghai University of Traditional Chinese Medicine, 1200 Cailun Road, Shanghai 201203, China; 2 Shanghai Skin Disease Hospital, Shanghai 200443, China; 3 Department of Anatomy and Cell Biology, College of Medicine, University of Florida, Gainesville, FL 32610, USA

**Keywords:** timosaponin AIII, glioblastoma, PDE5, cGMP pathway, β-catenin pathway

## Abstract

Glioblastoma (GBM) is the most aggressive brain tumor, with high mortality. Timosaponin AIII (TIA), a steroidal saponin isolated from the medicinal plant *Anemarrhena asphodeloides* Bge., has been shown to possess anticancer properties in various cancer types. However, the effect of TIA on GBM is unknown. In this study, we reveal that TIA not only inhibited U87MG in vitro cell growth but also in vivo tumor development. Moreover, we found that the cause of TIA-induced cell growth suppression was apoptosis. When seeking to uncover antitumor mechanisms of TIA, we found that TIA diminished the expression of cGMP-specific phosphodiesterase 5(PDE5) while elevating the levels of guanylate cyclases (sGCβ), cellular cGMP, and phosphorylation of VASP^ser239^. Following the knockdown of PDE5, PDE5 inhibitor tadalafil and cGMP analog 8-Bro-cGMP both inhibited cell growth and inactivated β-catenin; we reason that TIA elicited an antitumor effect by suppressing PDE5, leading to the activation of the cGMP signaling pathway, which, in turn, impeded β-catenin expression. As β-catenin is key for cell growth and survival in GBM, this study suggests that TIA elicits its anti-tumorigenic effect by interfering with β-catenin function through the activation of a PDE5/cGMP functional axis.

## 1. Introduction

Glioblastoma (GBM) is one of the deadliest malignant tumors in the central nervous system (CNS) [1]. The current treatments for GBM include surgical resection, radiotherapy, and chemotherapy with temozolomide. However, the benefit of these therapies to the survival of GBM patients is limited [2]. 

Cyclic 3′, 5′-GMP (cGMP) is a critical second messenger of cyclic nucleotides in cells. cGMP activates protein kinase G (PKG), and subsequent signaling pathways regulate a broad spectrum of physiological and pathological processes. The cGMP pathway has emerged as a therapeutic target in various cancers [3,4,5,6]. Based on the GBM-related miRNA–mRNA regulatory network, downregulated genes were found to be significantly enriched in the cGMP–PKG signaling pathway and calcium signaling pathway [7]. Given the tumor-suppressive role of the global miRNA system [8,9], we reason that the cGMP–PKG signaling pathway may be involved in negative regulation of GBM progression/development. 

The intracellular level of cGMP is determined by a dynamic balance between soluble guanylate cyclase (sGC)-mediated synthesis and cyclic nucleotide phosphodiester (PDEs)-led hydrolysis. Restoring sGC with an active sGC mutant (sGCα1β1(Cys105)) suppressed glioblastoma cell growth and increased the survival time in nude mice bearing a glioblastoma xenograft [10], which is consistent with the notion that the cGMP–PKG signaling pathway negatively regulates GBM progression/development.

Among cGMP-specific PDEs, phosphodiesterase 5 (PDE5) is expressed abnormally in a variety of cancers [11,12,13]. PDE5 inhibition reduces cancer stem cells’ maintenance via induction of PKA signaling [14], cancer cell growth, and tumor immunity through activating cGMP/PKG signaling to block Wnt/β-catenin transcription [15]. PDE5 inhibitors, which are safe drugs to treat erectile dysfunction, have shown benefits for cancer treatment [16,17]. For example, PDE5 inhibitor sildenafil inhibits colorectal cancer growth in vitro and in vivo [18], and administration of PDE5 inhibitors is reported to reduce the risk of colorectal cancer [19]. Moreover, PDE5 inhibitors increase the therapeutic efficacy of monoclonal antibodies to deter by effectively increasing blood–brain tumor barrier (BTB) permeability [20]. In addition, PDE5 inhibitor reversed BET inhibitor resistance in MYC/Ras-driven hepatocellular carcinoma [21] and sensitized docetaxel chemotherapy in prostate cancer cells [22]. These studies demonstrate that a persistent suppression of PDE5 may suppress steps critical for tumor progression, including tumor cell survival, stemness, and drug resistance. Therefore, identifying PDE5 inhibitors from natural medicinal plants for the development of anticancer drugs is of a great interest.

Rhizoma Anemarrhenae, the dried root of *Anemarrhena asphodeloides* Bge. (A.A.), which belongs to the family Liliaceae, has been widely used in China for centuries [23]. The chemical constituents of A.A. include steroidal saponins, flavones, xanthone C-glycosides, cyclic peptides, alkaloids, fatty acids, polyphenols, lignans, and polysaccharides. Numerous steroidal saponins have been found to exhibit diverse biological properties. In particular, timosaponin AIII (TIA, CAS no: 41059-79-4), a steroidal saponin with a sugar chain at the C3 position, presents a broad spectrum of biological activities including anti-inflammatory, anti-platelet and antithrombotic, anti-learning and memory deficits, and anticancer activities [24]. However, it is unknown whether TIA can suppress glioblastoma cell growth. 

In the present study, we show that TIA inhibited in vitro cell growth and in vivo xenograft development of U87MG cells. We present evidence that TIA elicits its tumorigenic effect by inactivating β-catenin via a PDE5/cGMP axis. 

## 2. Results

### 2.1. TIA Inhibited In Vitro Cell Growth and In Vivo Tumor Development of U87MG Cells

**Figure 1:**
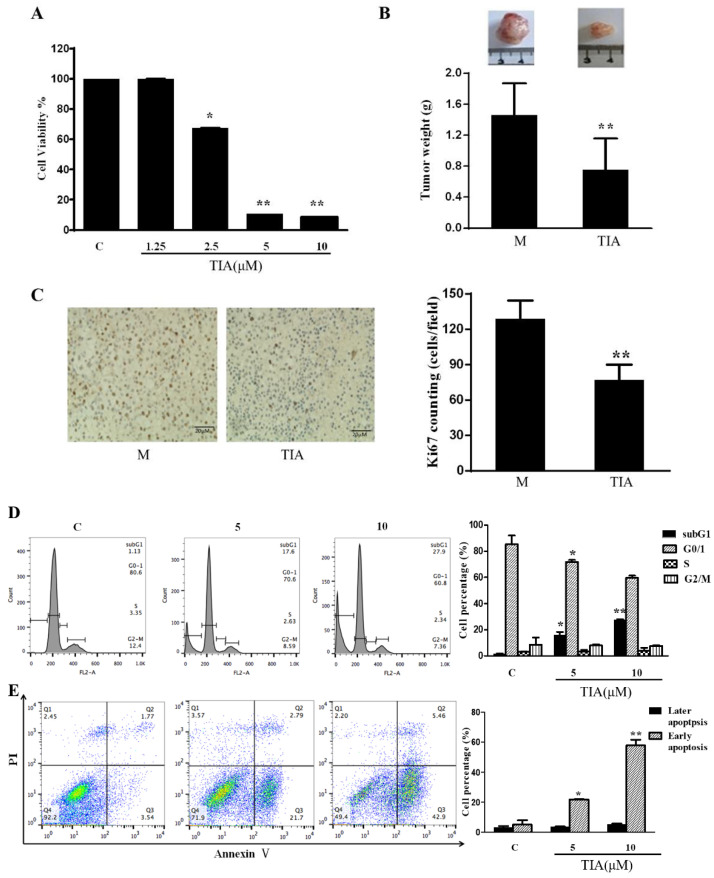
TIA suppresses U87MG cell growth by inducing apoptosis. (**A**) MTT assay to analyze the effect of TIA on U87 MG cell growth. (**B**) Effect of TIA on tumor weight. (**C**) IHC staining of Ki67 on tumors derived from mice treated with vehicle and TIA. (**D**) Effect of TIA on cell cycle progression of U87 MG cells. (**E**) Effect of TIA on apoptosis in U87MG cells. Data are the mean ± S.E.M. (*n* = 3). * *p* < 0.05 vs. controls; ** *p* < 0.01 vs. controls. C: control group of cells; 1.25–10: treatment of cells with 1.25–10 μM TIA; M: vehicle group of U87MG-bearing mice; TIA: oral administration of TIA (1 mg/kg/d, I.P., 14 days) to U87MG-bearing mice.

To determine the effect of TIA on glioblastoma cell growth, we treated U87MG cells with varying concentrations of TIA for 72 h. MTT assay showed that TIA dose-dependently inhibited U87MG cell growth (Figure 1A). To evaluate the effect of TIA on glioblastoma tumor development, we implanted U87MG cells in nude mice. After tumors reached sizes of approximately 200 mm^3^, at 2 weeks after implantation of cells, mice received daily TIA (1 mg/kg, I.P.) or a vehicle for two weeks. Compared to the vehicle group, treatment with TIA led to a 48% reduction in tumor weight (Figure 1B). Further immunohistochemistry staining revealed 40% less Ki67 staining in tumors excised from TIA-treated mice (Figure 1C). 

To understand the cause of TIA-elicited growth inhibition, we first analyzed the effect of TIA on the cell cycle progression of U87MG cells. Flow cytometry showed that TIA increased the percentage of the sub-G1 population (Figure 1D) while moderately decreasing the G0/1 cell population, indicating the occurrence of apoptosis. Subsequent annexin V/PI double-staining-based flow cytometry showed a dramatic increase in the early apoptotic cell population (Q3) in TIA-treated cells compared to the control (Figure 1E). These results suggested that TIA inhibited glioblastoma cell growth by inducing apoptosis.

### 2.2. TIA Downregulated the Abundance of β-Catenin, Cyclin D1, Bcl-2, and PDE5 in U87MG Cells

**Figure 2:**
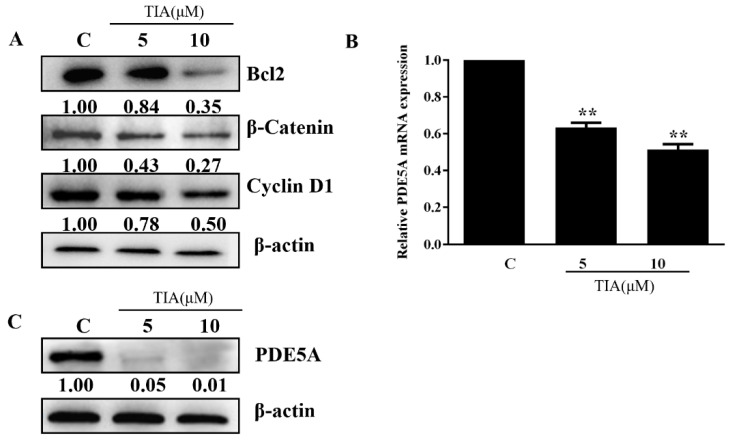
TIA is likely to downregulate the abundance of Bcl-2, β-catenin, and cyclin D1 through the suppression of PDE5. (**A**) Effects of TIA on the levels of Bcl-2, β-catenin, and cyclin D1 in U87 MG. (**B**,**C**). Effects of TIA on the mRNA (**B**) and protein (**C**) amounts of PDE5 in U87MG. Data are the mean ± S.E.M. (*n* = 3). ** *p* < 0.01 vs. controls. C: control group; 5: treatment with 5 μM TIA; 10: treatment with 10 μM TIA.

Apoptosis in glioblastoma has been reported to be closely associated with the abundance of β-catenin, cyclin D1 (a known β-catenin-regulated gene), and Bcl-2 [25]. To investigate if this is the case for TIA-induced apoptosis in U87MG cells, we treated cells with TIA with two different concentrations, 5 and 10 μM. Western blotting with the respective antibodies showed that TIA decreased the amounts of β-catenin, cyclin D1, and Bcl-2 in a concentration-dependent manner (Figure 2A). 

As activation of the β-catenin signaling pathway is known to be facilitated by PDE5 in various cancer types [26], we tested the potential involvement of PDE5 in this TIA-induced event and found that TIA reduced the expression of PDE5 (Figure 2B) while it almost completely wiped out the PDE5 protein in U87MG cells (Figure 2C). These results raised the possibility that TIA diminished PDE5, leading to the suppression of β-catenin, reduction in cyclin D1/Bcl-2, and subsequent apoptosis. 

**Figure 3:**
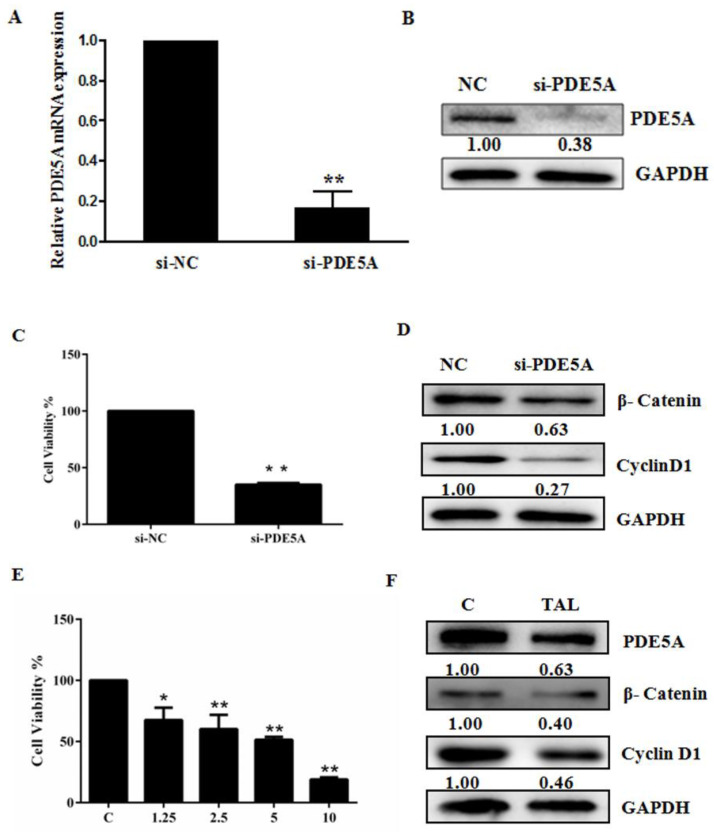
Interfering with PDE5A suppressed U87MG cell growth via the β-catenin pathway. (**A**,**B**) Effect of PDE5A siRNA on PDE5A mRNA (**A**) and protein (**B**) expression levels in U87 MG. (**C**–**F**) Effect of PDE5A knockdown and TAL inhibitor on cell growth and expression levels of PDE5, β-catenin, and cyclin D1 in U87 MG cells. Data are the mean ± S.E.M. (*n* = 3). * *p* < 0.05 vs. controls; ** *p* < 0.01 vs. controls. C: control group; 1.25–10: treatment with 1.25–10 μM TAL.

To test the effect of PDE5 on U87 MG cell growth, we initially introduced PDE5A siRNA into U87MG cells. Western blotting confirmed the effectiveness of PDE5 siRNA to silence PDE5A (Figure 3A). Compared to the control, the MTT assay showed that knockdown of PDE5 led to a 60% reduction in cell growth (Figure 3B). Moreover, we detected a marked reduction in the levels of β-catenin and cyclin D1 in PDE5A knockdown cells (Figure 3C). In a parallel experiment, we treated U87MG cells with PDE5 inhibitor tadalafil (TAL) for 24 h. MTT assay showed that TAL dose-dependently inhibited cell growth (Figure 3D). Similarly, TAL lessened the abundance of β-catenin and cyclin D1 (Figure 3E). Collectively, these results supported the notion that TIA inhibited glioblastoma cell growth by interfering with the PDE5–β-catenin signaling pathway.

### 2.3. Activation of the cGMP Pathway Inhibits U87MG Cell Growth by Interfering with β-Catenin

**Figure 4:**
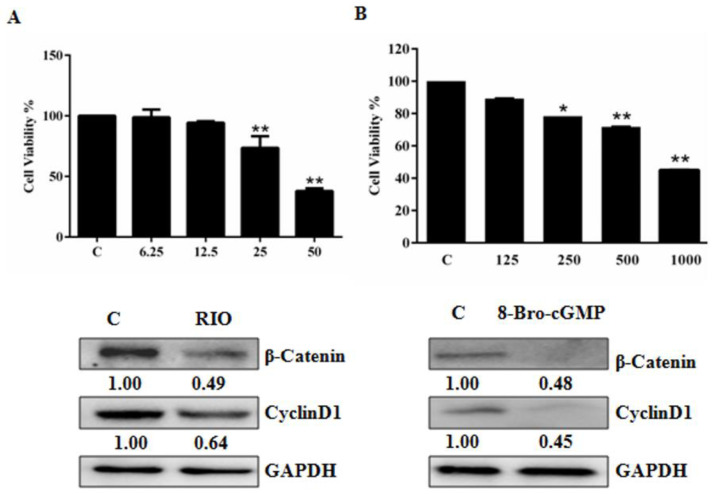
sGC stimulator RIO and cGMP analog 8-Bro-cGMP inhibited glioblastoma cell growth. (**A**) Effects of RIO and 8-Bro-cGMP on U87MG cell growth. (**B**) Effects of RIO and 8-Bro-cGMP on the abundance of β-catenin and cyclin D1. Data are the mean ± S.E.M. (*n* = 3). * *p* < 0.05 vs. controls; ** *p* < 0.01 vs. controls. C: control group; 6.25–50: treatment with 6.25–50 μM RIO; 125–1000: treatment with 125–1000 μM 8-bro-cGMP.

The nature of PDE5 as a cGMP decomposer prompted us to investigate the effect of the cGMP signaling pathway on U87MG growth. We treated U87MG cells with varying concentrations of 8-Bro-cGMP or sGC stimulator riociguat (RIO). MTT assay showed that both RIO (50 μM) and 8-Bro-cGMP (1000 μM) dramatically decreased the growth of U87MG cells (Figure 4A). Moreover, both RIO and 8-Bro-cGMP reduced the levels of β-catenin and cyclin D1 (Figure 4B).

**Figure 5:**
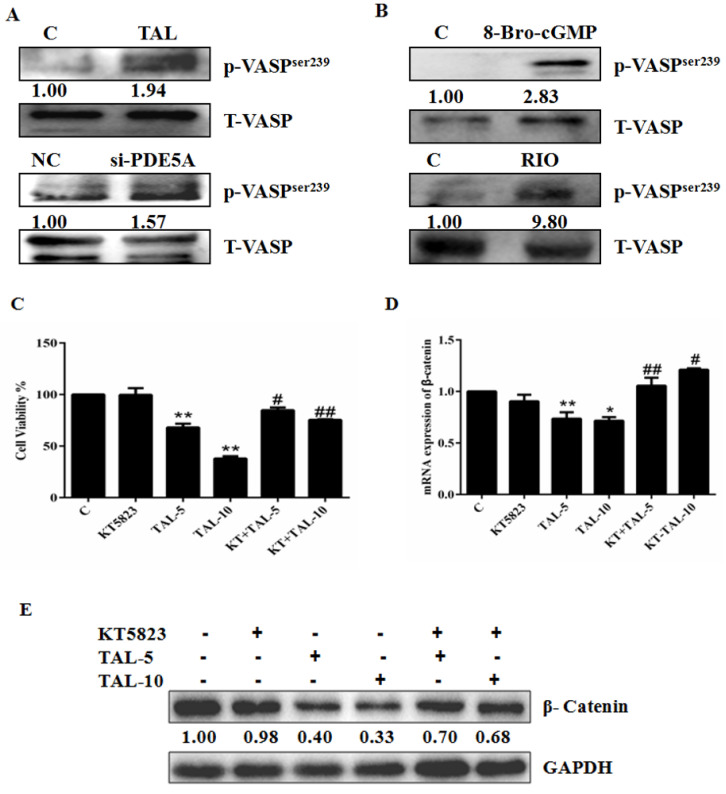
Activation of the cGMP pathway induces growth inhibition in glioblastoma cells. (**A**) Effect of TAL and si-PDE5 on the level of serine239-phosphorylated VASP in U87MG cells. (**B**) Effect of 8-Bro-cGMP and RIO on the level of serine239-phosphorylated VASP in U87MG cells. (**C**) Effects of KT5823 combined with TAL or alone on U87MG cell growth. (**D**) Effects of KT5823 combined with TAL or alone on mRNA expression of β-catenin. (**E**) Effects of KT5823 combined with TAL or alone on protein expression of β-catenin. Data are the mean ± S.E.M. (*n* = 3). * *p* < 0.05 vs. controls; ** *p* < 0.01 vs. controls. ^#^ *p* < 0.05 vs. TAL treatment alone; ^##^ *p* < 0.01 vs. TAL treatment alone. C: control group; KT5823: treatment with KT5823; TAL-5/TAL-10: treatment with TAL at 5 or 10 μM; KT+TAL-5/KT+TAL-10: treatment with KT5823 plus TAL at 5 or 10 μM.

Activation of protein kinase G, the effector of cGMP, induces apoptosis in colon cancer cells by blocking β-catenin expression [27,28]. To ensure that PDE5 inhibitor TAL, sGC stimulator RIO, and 8-Bro-cGMP exert their role through PKG, we assessed their effect on the status of serine239-phosphorylated VASP, a known site phosphorylated by PKG upon the elevation of cGMP. Western blotting showed that all three were able to increase the level of serine239-phosphorylated VASP in U87MG cells (Figure 5A,B), confirming the activation of the cGMP pathway in U87MG cells by TAL, RIO, or 8-Bro-cGMP. Subsequently, we pre-treated U87MG cells with KT 5823, a selective PKG inhibitor, followed by the addition of TAL. While KT5823 alone did little to impact cell growth, it essentially abolished growth inhibition elicited by TAL (Figure 5C). Parallel qPCR and Western blotting showed that KT5823 prevented TAL from decreasing the levels of both β-catenin mRNA and protein (Figure 5D,E). Together, these results suggest that activation of the cGMP pathway leads to cell growth suppression, partially by downregulating β-catenin abundance in U87MG. 

### 2.4. TIA Regulates the PDE5/cGMP Axis in U87MG Cells

**Figure 6:**
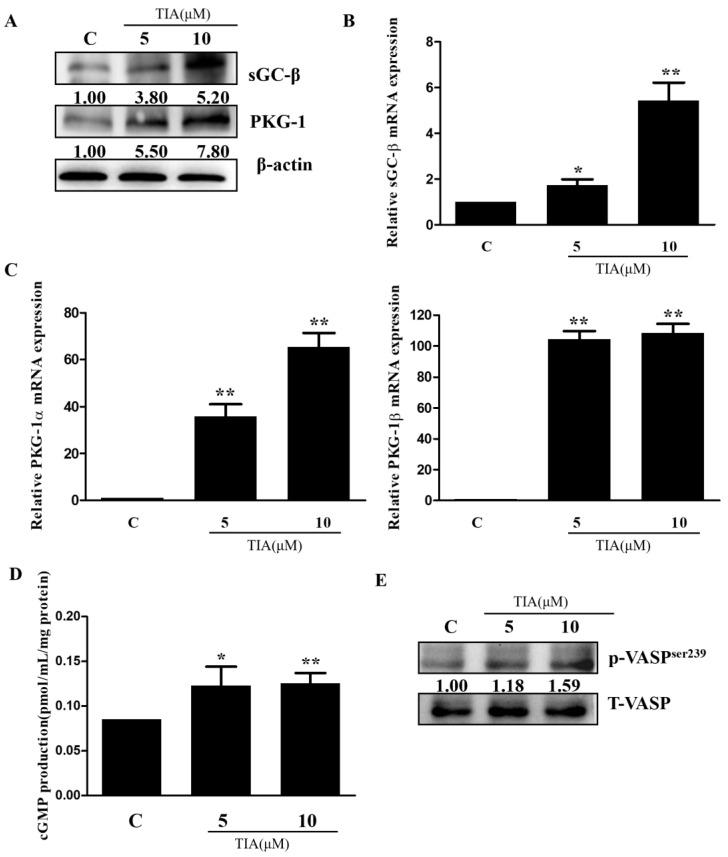
TIA activates the cGMP pathway in U87MG cells. (**A**–**C**) Effect of TIA on the mRNA and protein expression levels of sGCβ and PKG in U87MG. (**D**) Effect of TIA on the cellular level of cGMP in U87MG cells. (**E**) Effect of TIA on the phosphorylation level of vaspser239 protein in U87MG cells. Data are the mean ± S.E.M. (*n* = 3). * *p* < 0.05 vs. controls; ** *p* < 0.01 vs. controls. C: control group; 5: treatment with 5 μM TIA; 10: treatment with 10 μM TIA.

The ability of TIA to downregulate the abundance of PDE5 prompted us to determine whether TIA also affects other elements in the cGMP pathway. qPCR and Western blotting showed that TIA dose-dependently increased the abundance of sGCβ and PKG (Figure 6A–C). Furthermore, TIA nearly doubled the cellular cGMP concentration (Figure 6D) and increased the level of serine239-phosphorylated VASP in U87 glioma cells (Figure 6E). These results indicate that TIA inhibits cell growth by regulating multiple vital components in the cGMP signaling pathway, including downregulation of PDE5 and upregulation of sGCβ/PKG to elevate the cGMP level. 

## 3. Discussion

Active ingredients isolated from traditional medical herbs are considered promising and potentially valuable resources for developing anticancer drugs. TIA has recently received particular attention for its potential to be developed as an anticancer agent [29] because TIA has been demonstrated to exert potent and diverse antitumor activities including interfering with invasion and migration [30,31,32,33,34], autophagy [35,36,37], reversing drug resistance [38,39,40,41], and inducing apoptosis [42,43,44,45,46,47]. However, the effect of TIA on glioblastoma tumorigeneity remains unanswered. In this study, we presented evidence that TIA significantly inhibited cell growth by arresting cell cycle progression and inducing apoptosis in human glioblastoma cell U87MG. Importantly, TIA effectively deterred tumor development in a mouse xenograft model (Figure 1).

Previous studies have linked multiple signaling pathways to TIA-led growth inhibition. For instance, TIA was proposed to interfere with mTOR function and induce ER stress in breast and prostate cancer cells [42]. It was reported to trigger mitochondria-mediated and caspase-dependent apoptosis in liver cancer cells [43]. In human melanoma cells, TIA was noted to activate c-Jun N-terminal protein kinase (JNK) and the extracellular-signal-related kinase (ERK) signaling pathway, leading to the production of NO [37]. Moreover, TIA was shown to intercept the PI3K/Akt and STAT3 pathways in pancreatic cancer cells [44,45], diminish ERK1/2 activity in lung cancer cells [46], and activate the ATM/Chk2 and p38 MAPK pathways in breast cancer [47]. In this study, we provided evidence that TIA potently stimulated the cGMP–PKG signaling pathway in GBM cells. We speculate that TIA may target diverse signaling pathways depending on the cell types. 

A previous bioinformatics study proposed that the cGMP–PKG signaling pathway may negatively contribute to GBM progression [7]. Our study showed that knockdown of PDE5, treatment with PDE5 inhibitor tadalafil, activation of sGC by RIO, and directly increasing the intracellular cGMP concentration all hampered U87MG cell growth (Figure 3, Figure 4 and Figure 5). We believe that our study provides the first experimental data supporting the role of the cGMP–PKG signaling pathway in GBM cell growth and tumor development. 

A possible mechanism underlying the antitumor role of the cGMP pathway is the functional connection between the cGMP and β-catenin signaling pathways. Activated cGMP signaling blocks β-catenin transcription, leading to cancer cell death [14,16]. PKG activation has also been shown to interfere with the β-catenin signaling pathway by suppressing the nuclear translocation of β-catenin, reducing β-catenin expression, and decreasing the transcriptional activity of β-catenin [27,28]. These previous observations are consistent with our data, which revealed that blocking PDE5 function (either using PDE5 inhibitor TAL or PDE5A siRNA) and 8-Bro-cGMP not only reduced the level of β-catenin and but also its target gene cyclin D1 in U87MG (Figure 3, Figure 4 and Figure 5). In addition, our findings suggest that activation of the cGMP–PKG signaling pathway may represent an effective strategy to suppress the oncogenic activity of β-catenin and tumor development in GBM.

**Figure 7:**
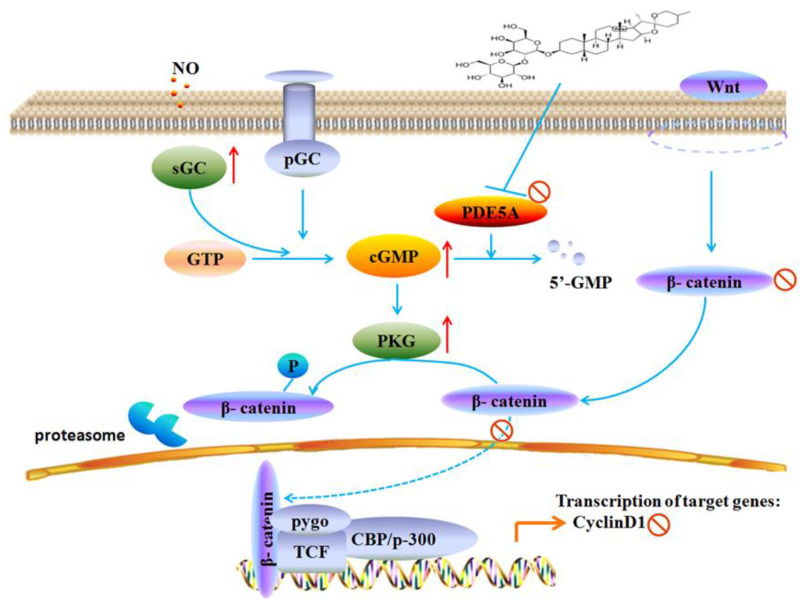
The potential mechanism of TIA to impact U87MG growth.

In conclusion, this study demonstrates that TIA can effectively inhibit GBM cell growth and tumor development. Mechanically, TIA exerts its action by downregulating cGMP-specific PDE5 and upregulating sGC, leading to the accumulation of intercellular cGMP and subsequent activation of the PKG signaling pathway (Figure 6). Activation of PKG further blocked β-catenin function, resulting in the obstruction of GBM cell growth and tumor development (Figure 7).

## 4. Materials and Methods

### 4.1. Reagents

Timosaponin AIII (TIA) was purchased from Tauto Biotech Co. (41059-79-4, with purity >98% by HPLC, Shanghai, China); U87MG was obtained from the American Tissue Culture Collection (Manassas, VI, USA); DMEM and FBS were obtained from HyClone (Auckland, New Zealand); MTT and DMSO were obtained from Sigma (St. Louis, MO, USA); a high-capacity cDNA reverse transcription kit, RNAIMAX transfect reagent, and TRIzol reagent were purchased from Thermo Fisher Scientific Company (Waltham, MA, USA); RealMasterMix (SYBR Green), protease inhibitor cocktail, and PhosSTOP were obtained from Roche (Basel, Switzerland); a protein marker was purchased from Promega (Madison, WI, USA); nitrocellulose membrane was obtained from GE Whatman (GE Healthcare Bio-Sciences, Pittsburgh, PA, USA); β-actin, β-catenin, PKG1, T-VASP, and p-VASPser239 antibodies were purchased from Cell Signaling Technology (Danvers, MA, USA); PDE5A, sGCβ, cyclin D1 antibodies, goat anti-mouse IgG H&L, goat anti-rabbit IgG H&L, and 8-Bro-cGMP were purchased from Abcam (Cambridge, MA, USA). An ECL chemiluminescence kit was purchased from Millipore (Burlington, MA, USA); the Bcl-2 antibody, BCA protein assay kit, RIPA protein lysate, and cell cycle analysis kit were obtained from Beyotime Institute of Biotechnology (Haimen, China); the FITC/annexin V apoptosis detection kit was provided by BD Biosciences (San Jose, CA, USA); riociguat was acquired from (Meilune, Dalian, China), the cGMP EIA kit was obtained from Cayman (Ann Arbor, MI, USA); tadalafil was acquired from Santa Cruz (Dallas, TX, USA); the siRNA negative control and siRNA-PDE5A were synthesized by GenePharm (Shanghai, China), with 5′-GGAAGAAACAAGAGAGCUAdTdT-3′ as the sequence of the latter; and all PCR primers were synthesized by Bioengineering Co., Ltd. (Shanghai, China).

### 4.2. Cell Culture and Transfection

U87MG cells were maintained in DMEM medium supplemented with 10% FBS at 37 °C in a humidified 5% CO_2_ atmosphere. After a stable 2–3 passages, cells in the logarithmic growth phase were used for experiments.

The siRNAs were transfected into cells using RNAIMAX (Invitrogen, Waltham, MA, USA) as instructed by the manufacturer. The cells were subjected to gene and protein evaluations after 72 h of treatments.

### 4.3. Cell Proliferation Assay

The U87MG cell suspension was adjusted to a density of 5 × 10^4^/mL and seeded in 100 μL of each well in a 96-well plate overnight, followed by treatment with TIA (1.25–10 µM), TAL (0.125–2 μM), 8-Bro-cGMP (125–1000 μM), or RIO (6.25–50 μM) for 24 h. MTT solution was added to each well for 4 h, and the cell number in each well was determined by measuring the absorbance of DMSO-solubilized crystals at 490 nm using a microplate reader. Cell growth inhibition was calculated using the formula of (OD-treated group mean − OD blank control group mean)/(OD control group mean − OD blank control group mean) × 100%.

### 4.4. Cell Cycle and Apoptosis Analyses

Cell cycle analysis was performed using FACS analysis with propidium iodide (PI) staining. U87MG cells were starved in a serum-free medium for 18 h and then treated with various concentrations of TIA for 6 h. Cells were washed and then fixed with 70% ethanol at 4 °C for 24 h. Fixed cells were washed and then incubated for 30 min with RNase (μg/mL) and PI (μg/mL), followed by flow cytometry. Cell cycle distribution was analyzed with 10,000 collected cells using CellQuest acquisition and the Flowjo analysis program (v10.6.2). Apoptosis was analyzed using an FITC/annexin V apoptosis detection kit according to the manufacturer’s protocol, and approximately 10,000 gated events were analyzed for each treatment.

### 4.5. qRT-PCR

U87MG cells were cultured overnight and then replaced by fresh serum-free DMEM culture medium. TIA (5/10 μM) was added to the treatment groups, which then continued to cultivate for 4 h, before 1 mL TRIzol was added to each group in accordance with the TRIzol instructions for the extraction step. The reaction conditions were: 25 °C × 10 min, 37 °C × 120 min, and 85 °C × 5 min. The products were stored at 4 °C, according to the instruction of the high-capacity reverse transcription kit. qRT-PCR was performed by synthesizing template cDNAs in a 20 μL reaction system according to the RealMasterMix (SYBR Green) protocol. The primers were as follows: GUCY1B3 forward 5′-GGAAATTGCTGGCCAGGTTCAAGT-3′; reverse 5′-TTCTCCTGTGGTTTCTGTTCGGCT-3′; PDE5A forward 5′-GAAGCATGGCTGGACGATCA-3′; reverse 5′-AGGGGCACTGTTATCTGCAC-3′; PKG1α forward 5′-GGCTGTCAGAGAAGGAGGAAG-3′; reverse 5′-GGAAGGACCTGTACGTCTGC-3′; PKG1β forward 5′-GCACCTTGCGGATTTACAG-3′; reverse 5′-TTCTGGATCTCGTCCTTCTG-3′; β-actin forward 5′-CTCCTCCTGAGCGCAAGTACTC-3′; reverse 5′-CGGACTCGTCATACTCCTGCT-3′. The conditions were as follows: 95 °C × 10 min, 50 °C × 2 min, 95 °C × 15 s, and 60 °C × 1 min (40 cycles). Three replicates were conducted for each sample. The relative content of the TIA treatment group was calculated using 2^−ΔΔ^Ct.

### 4.6. Western Blotting Analysis

The cells were divided into different groups with particular treatments. After washing with iced PBS, the cells were harvested by adding phosphatase inhibitor, protease inhibitor, and RIPA buffer on ice, and then centrifuged at 4 °C to 12,000× *g* rpm for 15 min. The protein concentrations of the clarified supernatants were determined by a BCA protein assay kit. Then, 30 μg protein was resolved on 10% SDS-PAGE and transferred to PVDF membranes. These membranes were blocked with 5% nonfat dry milk for 1 h and incubated in primary antibodies against β-actin, Bcl-2, β-catenin, cyclin D1, PDE5A, PKG1, sGCβ, T-VASP, and p-VASP^ser239^ in 5% BSA solution, and then incubated overnight at 4 °C. Following several washes, the membranes were incubated with the corresponding secondary antibody. Next, the membranes were washed with TBST for 5 min × 6 times. After that, the blots were developed using an ECL kit.

### 4.7. cGMP Analysis

To determine the concentration of cGMP in U87MG cells and the effect of timosaponin AIII on the concentration, cells were plated at a density of 1 × 10^6^ cells per 100 mm plate and were treated with TIA or the vehicle control. After 45 min of treatment, we aspirated the medium from the plate and added 150 μL of 0.1 M HCL per plate. We decanted the supernatant after centrifuging 1000 g for 10 min. The cGMP in supernatants was quantified by a colorimetric competitive cGMP EIA kit. The assay was carried out according to the manufacturer’s specifications.

### 4.8. Animals and Xenograft Studies

Male BALB/C athymic nude mice (six-week-old, with an initial body weight of 20–22 g) were purchased from SLAC Laboratory Animal Co., Ltd. (Shanghai, China) and housed under sterile conditions with controlled temperature (22 °C), humidity, and a 12 h light/dark cycle.

The mice were inoculated with 2 × 10^6^ cells/100 μL into the flank. Tumor dimensions were measured twice a week, and the volume was calculated as length × width^2^ × 0.52. When tumors reached ~200 mm^3^, mice were treated with TIA (1 mg/kg/d, I.P.) for 14 days, and then the mice were sacrificed.

### 4.9. Immunohistochemistry

Tumors were excised in 4 mm sections, fixed with formalin, and selected samples were embedded with paraffin. Slides were stained with antibodies against ki67 (at dilutions of 1:100) and then, after washing, stained with secondary antibody. Finally, the stained sections were analyzed under a microscope at a magnification of ×400.

### 4.10. Statistics

Student’s *t*-test or ANOVA was used where appropriate. *p*-values < 0.05 were considered significant. Experiments were performed with a minimum of three replicates.

## 5. Conclusions

In summary, we conclude that TIA inhibited U87MG cell proliferation and induced apoptosis by suppressing the expression of PDE5, increasing the amount of sGCβ, restoring intracellular cGMP levels, and activating PKG, the essential downstream proteins of cGMP. The activation of the cGMP pathway suppressed U87MG cell growth via its ability to block the β-catenin pathway.

## Data Availability

The datasets used and/or analyzed during the current study are available from the corresponding author upon reasonable request.

## Sample Availability

Samples of the compounds are available from the authors.
